# Whipple’s Disease Manifested as Recurrent Ascites

**DOI:** 10.7759/cureus.1108

**Published:** 2017-03-21

**Authors:** Ali Aamar, Kamraan Madhani, Muhammad S Anwar, Prabhdeep Singh, Joel Garsten

**Affiliations:** 1 Internal Medicine, Yale Waterbury; 2 Gastroenterology, Waterbury Hospital

**Keywords:** whipple’s disease, ascites, small intestine

## Abstract

Whipple’s disease commonly presents as chronic diarrhea and abdominal pain. Ascites is an uncommon presentation of Whipple’s disease. Here, we report a rare case of a 47-year-old male who presented with diarrhea and abdominal distention for three months. The physical examination was significant for ascites. Serum albumin was low and serum-to-ascites albumin gradient was < 1.1 g/dl. This suggested that ascites was less likely to be present due to portal hypertension. Enteroscopy showed erythematous duodenum and jejunum; biopsy was suggestive of periodic acid-Schiff stain (PAS) positive macrophages consistent with Whipple’s disease. During the hospital course, the patient improved with intravenous (IV) ceftriaxone.

## Introduction

Whipple’s disease is caused by Tropheryma whipplei*,* a gram-positive bacillus related to actinomycetes. The disease was first described by George H. Whipple in 1907. Whipple’s disease is a rare multisystem bacterial infection that primarily affects the small intestine [1]. A delay in diagnosis can be fatal due to multisystem involvement. Whipple’s disease commonly presents as chronic diarrhea but it rarely manifests as recurrent ascites. The typical clinical manifestations of Whipple's disease are chronic diarrhea, weight loss, and abdominal pain [2]. We describe a rare case of Whipple’s disease that presented with diarrhea and recurrent ascites.

## Case presentation

**Figure 1:**
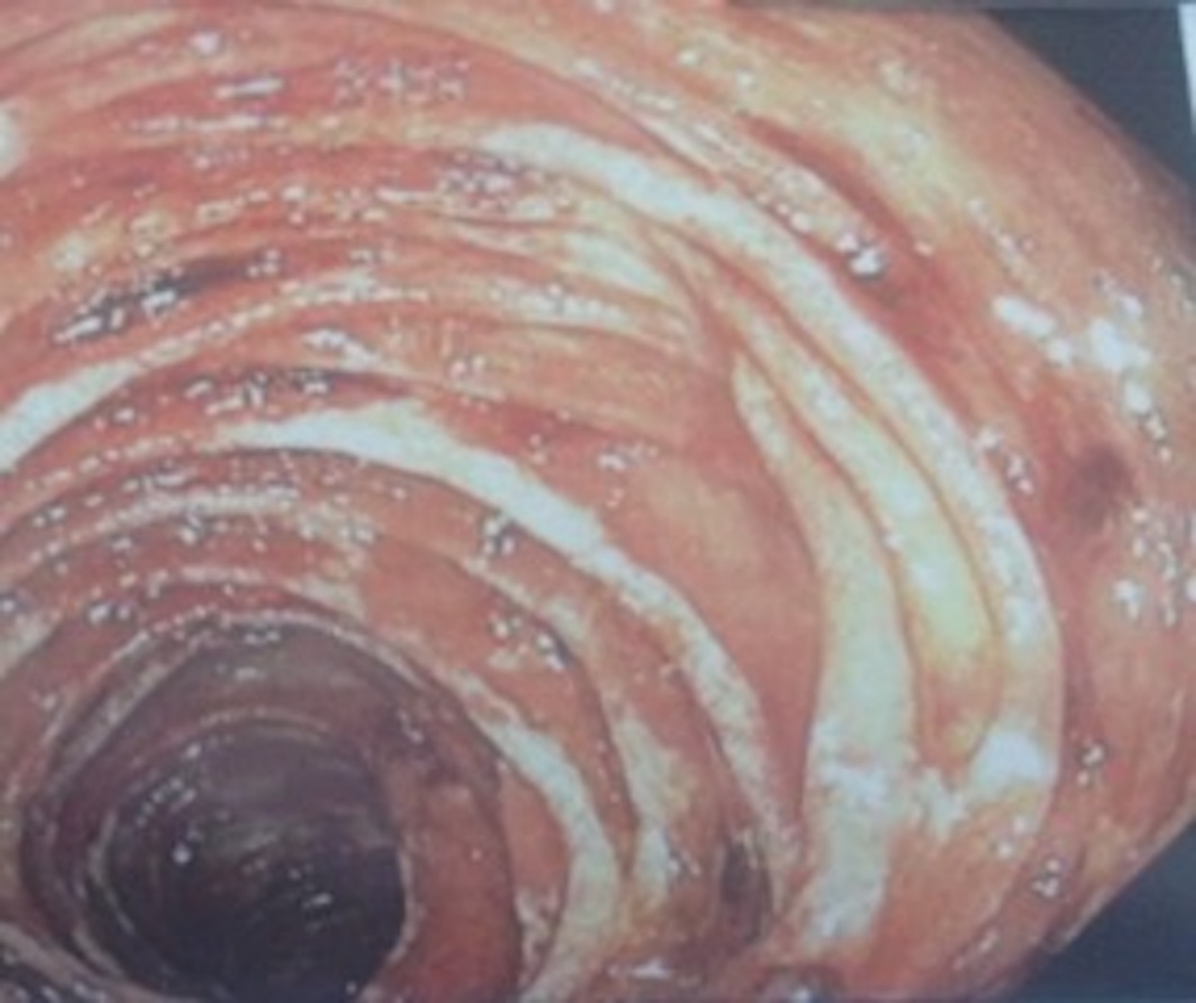
Small Bowel Endoscopy Small bowel endoscopy showing erythematous and edematous mucosa.

A 47-year-old male presented with diarrhea and a worsening abdominal distention for three months. The physical examination was remarkable for muscle wasting and ascites. Laboratory analysis showed hemoglobin 7.2 g/dl, hematocrit 22.7%, mean corpuscular volume (MCV) 77.3 fl, platelets 172 thousand/mm3, serum albumin 1.9 g/dl, total protein 4.1 g/dl, bilirubin 0.3 mg/dl, alanine transaminase (ALT) 23 IU/L, aspartate aminotransferase (AST) 28 IU/L, international normalized ratio (INR) 1.2, iron 23 mcg/dl, and ferritin 24 ng/ml. Stool analysis was negative for blood, clostridium difficile, ova, and parasites. Urine analysis was negative for protein. Hepatitis viral serologies, immunoGlobulins A anti-tissue transglutaminase antibody (IgA-anti-tTG), antinuclear antibody (ANA), anti-mitochondrial antibody (AMA), and anti-smooth muscle antibodies (AMSA) were all negative. Alpha-1 antitrypsin and ceruloplasmin levels were normal. Ascitic fluid was clear with albumin 1.1 g/dl, protein 2.9 g/dl, and white blood cell (WBC) 63/mm3 with two percent granulocytes and 17% lymphocytes. Ascitic fluid was negative for any malignant cells. Serum-to-ascites albumin gradient was < 1.1 g/dl; therefore, ascites was less likely to be present due to portal hypertension. Echocardiography (ECG) showed ejection fraction of 60-65% with a pulmonary artery systolic pressure of 42 mmHg. Right heart catheterization showed mild pulmonary hypertension. The severity of ascites could not be explained by mild pulmonary hypertension. Upper gastrointestinal (GI) endoscopy and colonoscopy were normal. Therefore, no biopsies were performed. He had recurrent ascites that was managed periodically with therapeutic paracentesis and diuretics. After eight weeks, the patient became severely malnourished and he was started on total parenteral nutrition. As recurrent ascites could not be explained by mild pulmonary hypertension, a liver biopsy was performed. The liver biopsy was normal. Enteroscopy showed the erythematous, edematous duodenum and jejunum (Figure 1).

**Figure 2:**
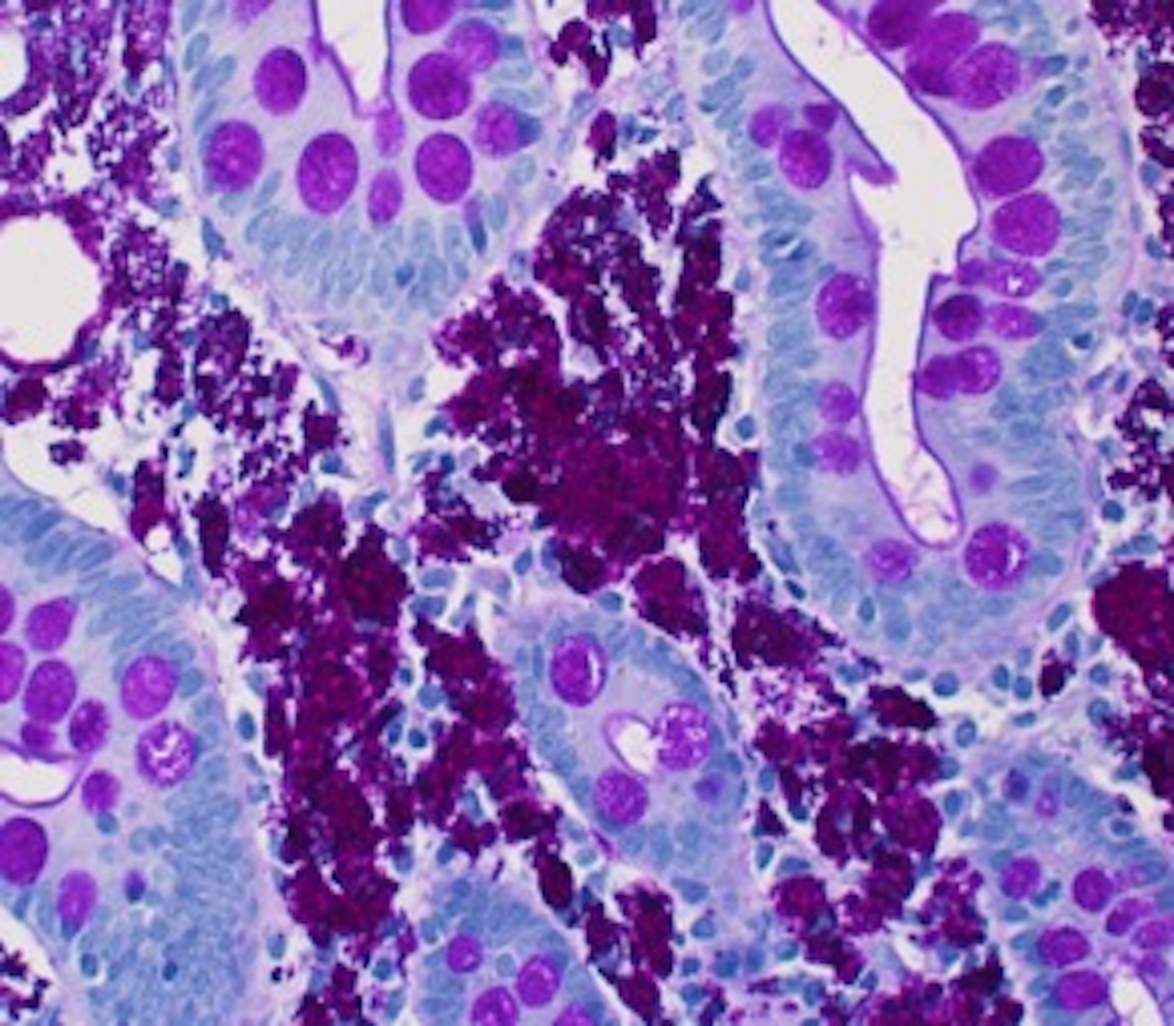
Small Bowel Biopsy Small bowel biopsy showing PAS positive macrophages in lamina propria.

The duodenal bulb was erythematous, which was normal in the initial upper GI endoscopy. Biopsy samples from the inflamed mucosa showed abundant periodic acid-Schiff stain (PAS) positive macrophages consistent with Whipple’s disease (Figure 2).

The patient was started on intravenous (IV) ceftriaxone. During the hospital course, the patient’s diarrhea improved on ceftriaxone and he was discharged on a one-year course of co-trimoxazole.

## Discussion

Whipple’s disease is a rare multisystem bacterial infection. Our case report aims to highlight ascites as an uncommon manifestation of Whipple's disease. The disease has an annual incidence of less than one per million. It is more common in middle-aged white men [3]. Even though the causative bacterium is ubiquitously present in the environment, the risk of infection is rare. Occupational exposure to soil and animals increases the risk of infection [3].

The classic presentation of Whipple’s disease is characterized by arthralgias (80%), diarrhea (76%), abdominal pain (55%), and weight loss (92%). Some patients have severe symptoms of malabsorption, such as ascites (eight percent) and peripheral edema [4]. Involvement of the central nervous system (CNS) was reported in 10% to 40% of the cases. Neurological involvement can present as cognitive dysfunction, dementia, memory impairment, cerebellar ataxia, and abnormal ocular movements [5]. Cardiac involvement can manifest as culture-negative endocarditis. Pulmonary hypertension has been associated with Whipple’s disease in a few case reports for which the underlying pathophysiological mechanism is unclear. Our patient also had mild pulmonary hypertension, as suggested by right heart catheterization. The strongest evidence of the causal relationship of pulmonary hypertension with the disease is its reversibility with antibiotics [6].

Upper GI endoscopy and biopsy of the small intestine are the diagnostic tests of choice. The endoscopic appearance is described as pale plaques alternating with erythematous and friable mucosa [7]. The main histological features are PAS positive macrophages in lamina propria and villous atrophy. The duodenal lesions can be focal; multiple biopsies should be studied when diagnosis is suspected. Polymerase chain reaction (PCR) testing for the causative organism has a 97% sensitivity and a 100% specificity [8]. Electron microscopy may be required for non-intestinal tissue involvement [8].

Whipple’s disease is treated by initial therapy with ceftriaxone or penicillin G for two weeks followed by trimethoprim-sulfamethoxazole for one year [9]. Patients who are allergic to penicillin can use meropenem for the initial intravenous course [10]. Clinical improvement is rapid, occurring in seven to twenty-one days. The response to treatment can be monitored by the resolution of symptoms and improvements in weight and hematocrit [10].

## Conclusions

Whipple's disease is rare and ascites is an even more uncommon presentation of the disease. Reporting these cases can help us better understand these rare manifestations of Whipple's disease. Whipple’s disease is a multisystem, bacterial infection caused by Tropheryma whipplei,* *primarily affecting the small intestine. Whipple’s disease should be considered in the differential diagnosis of chronic diarrhea and ascites. Whipple’s disease is treatable with a two-week course of IV ceftriaxone followed by co-trimoxazole for one year.
